# Domatinostat Targets the FOXM1–Survivin Axis to Reduce the Viability of Ovarian Cancer Cells Alone and in Combination with Chemotherapeutic Agents

**DOI:** 10.3390/ijms241310817

**Published:** 2023-06-28

**Authors:** Yurika Nakagawa-Saito, Yuta Mitobe, Shuhei Suzuki, Keita Togashi, Asuka Sugai, Chifumi Kitanaka, Masashi Okada

**Affiliations:** 1 Department of Molecular Cancer Science, School of Medicine, Yamagata University, 2-2-2 Iida-Nishi, Yamagata 990-9585, Japan; 2 Department of Neurosurgery, School of Medicine, Yamagata University, 2-2-2 Iida-Nishi, Yamagata 990-9585, Japan; 3 Department of Clinical Oncology, School of Medicine, Yamagata University, 2-2-2 Iida-Nishi, Yamagata 990-9585, Japan; 4 Department of Ophthalmology and Visual Sciences, School of Medicine, Yamagata University, 2-2-2 Iida-Nishi, Yamagata 990-9585, Japan; 5 Research Institute for Promotion of Medical Sciences, Faculty of Medicine, Yamagata University, 2-2-2 Iida-Nishi, Yamagata 990-9585, Japan

**Keywords:** drug repositioning, drug repurposing, gynecological malignancy, epigenetic therapeutics

## Abstract

The deregulation of the FOXM1 transcription factor is a key molecular alteration in ovarian cancer, contributing to the development and progression of ovarian cancer via activation of the target genes. As such, FOXM1 is a highly attractive therapeutic target in the treatment of ovarian cancer, but there has been no clinically tested FOXM1 inhibitor to date. We investigated in this study the effects of domatinostat, a class I-selective HDAC inhibitor currently in the clinical stage of development as a cancer therapeutic, on the expression of FOXM1 and viability of ovarian cancer cells. Cell viability, as well as protein and mRNA expression of FOXM1 and its transcriptional target survivin, was examined after domatinostat treatment of TOV21G and SKOV3 ovarian cancer cell lines in the absence or presence of cisplatin and paclitaxel. The effect of FOXM1 knockdown on survivin expression and those of genetic and pharmacological inhibition of survivin alone or in combination with the chemotherapeutic agents on cell viability were also examined. Domatinostat reduced the protein and mRNA expression of FOXM1 and survivin and also the viability of ovarian cancer cells alone and in combination with cisplatin or paclitaxel at clinically relevant concentrations. Knockdown experiments showed survivin expression was dependent on FOXM1 in ovarian cancer cells. Survivin inhibition was sufficient to reduce the viability of ovarian cancer cells alone and in combination with the chemotherapeutic agents. Our findings suggest that domatinostat, which effectively targets the FOXM1–survivin axis required for the viability of ovarian cancer cells, is a promising option for the treatment of ovarian cancer.

## 1. Introduction

Ovarian cancer, the eighth most common cancer and leading cause of cancer-related deaths in women worldwide, is among the deadliest of all gynecological malignancies, with the 5-year survival rate hovering around 50% [1,2]. Cytoreductive surgery followed by platinum- and taxane-based chemotherapy has been the mainstay of treatment for ovarian cancer; however, chemoresistance and recurrence after chemotherapy very frequently occur and lead to treatment failure. Although the recent introduction of poly (ADP-ribose) polymerase inhibitors has provided a significant improvement in patients’ outcomes, the prognosis of ovarian cancer nevertheless remains dismal, underscoring the need to elucidate the mechanisms behind its therapy-resistance and thereby develop novel therapies [1,3].

In an attempt to illuminate the whole picture of molecular alterations associated with ovarian cancer, a large-scale study was organized to conduct integrated genomic analyses of ovarian cancer, which highlighted FOXM1 as a key molecule, demonstrating the deregulation of the FOXM1 transcription factor network in nearly 90% of ovarian cancer cases [4]. Importantly, not only is FOXM1 associated with tumor progression and poor prognosis of ovarian cancer, but a large body of evidence from functional studies so far has established the pivotal roles of FOXM1 in cellular proliferation, migration, invasion, and chemoresistance of ovarian cancer [5], suggesting that FOXM1 is a rational and attractive therapeutic target for ovarian cancer, in particular for aggressive ovarian cancer with poor prognosis. Indeed, the potential of existing FOXM1 inhibitors as a therapeutic against ovarian cancer has been actively explored both in vitro and in vivo and, at the same time, the development of novel inhibitors of FOXM1 intended particularly for use in the treatment of ovarian cancer is enthusiastically pursued, with some being reported just recently [5,6,7]. However, none of them have yet to be tested in humans [8], and their safety in humans is therefore unknown.

Domatinostat (4SC-202) is an orally available, class I-selective small molecule inhibitor of HDAC, which has shown acceptable tolerability and safety in humans in a phase I study [9]. The anti-cancer activity of domatinostat has been tested and demonstrated in vitro and in vivo in a number of cancer types but not in ovarian cancer. Intriguingly, previous studies indicated that domatinostat reduced the expression of FOXM1 in pancreatic cancer and atypical teratoid/rhabdoid tumor cells [10,11]. Furthermore, we also observed that domatinostat reduced FOXM1 expression in glioma stem cells [12]. These findings suggested the possibility that domatinostat has the capacity to therapeutically target FOXM1 in ovarian cancer cells and suppress their malignant phenotypes.

To test this possibility, we determined in this study how domatinostat impacts the FOXM1 pathway and cellular properties associated with the pathway, such as proliferation, survival, and sensitivity to chemotherapeutic agents, in ovarian cancer cells.

## 2. Results

### 2.1. Domatinostat Reduces the Expression of FOXM1 in Ovarian Cancer Cells

**Figure 1:**
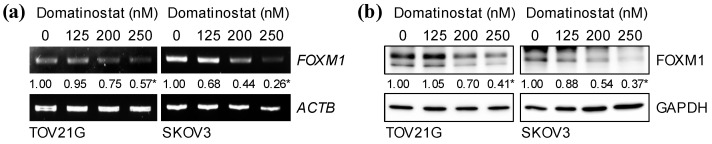
Domatinostat reduces the mRNA and protein expression of FOXM1 in ovarian cancer cells. Ovarian cancer cells (TOV21G and SKOV3) were treated with the indicated concentrations of domatinostat. After 3 days, the treated cells were subjected to RT-PCR (**a**) or Western blot (**b**) analysis for FOXM1 expression. Shown are representative images of two independent biological replicates. The numbers below the RT-PCR and Western blot images represent the means of the relative band intensities after each band was quantified by densitometry and normalized by the *ACTB* (RT-PCR) or the GAPDH (Western blot) value. * *p* < 0.05 vs. cells treated without domatinostat (i.e., at 0 nM).

Ovarian cancer is a spectrum of malignancies with several different histological profiles, of which serous, clear cell, and endometrioid ovarian carcinomas are recognized as the three major histotypes [3]. Although FOXM1 was originally identified as being frequently deregulated in serous ovarian carcinomas, the predominant histotype [4], a subsequent study demonstrated that FOXM1 was similarly overexpressed in non-serous (clear cell and endometrioid) ovarian carcinomas as well as in serous ones [13]. Notably, the study also revealed that FOXM1 overexpression was an independent indicator of worse disease-specific survival in non-serous rather than in serous cases, suggesting that FOXM1 might have a more dominant role in dictating the clinical aggressiveness in non-serous ovarian carcinomas than in serous ones. In the following experiments, therefore, we used both clear cell (TOV21G) and serous (SKOV3) ovarian cancer cell lines and examined the effects of domatinostat on these cell lines. First of all, we asked whether domatinostat could inhibit the expression of FOXM1 in ovarian cancer as it did in other cancer types [10,11,12]. When the ovarian cancer cell lines were treated with domatinostat at concentrations within the clinically relevant range, the expression levels of FOXM1 mRNA and protein decreased in a concentration-dependent manner (Figure 1a,b), suggesting that domatinostat inhibited FOXM1 expression at the transcriptional level.

### 2.2. Domatinostat Inhibits the Growth and Survival of Ovarian Cancer Cells

**Figure 2:**
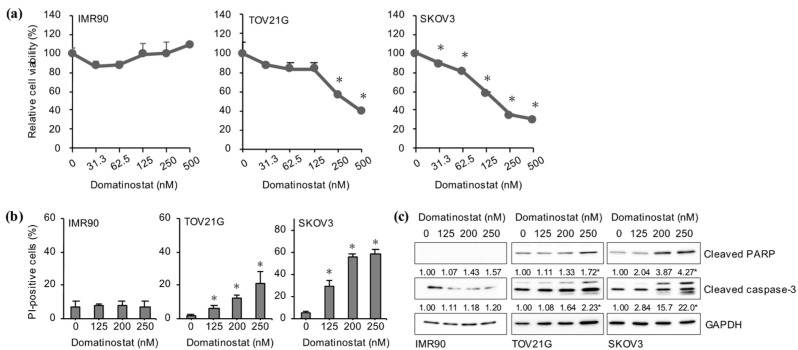
Domatinostat inhibits the growth and survival of ovarian cancer cells. (**a**) Normal human fibroblasts (IMR90) and ovarian cancer cells (TOV21G, SKOV3) were treated with the indicated concentrations of domatinostat for 3 days and then subjected to the WST-8 assay. Values are presented as the means + SD of triplicate samples. * *p* < 0.05 vs. cells treated without domatinostat (i.e., at 0 nM). (**b**) Cells were treated with the indicated concentrations of domatinostat for 3 days and then subjected to the propidium iodide (PI) incorporation assay. Values are presented as the means + SD of triplicate samples. * *p* < 0.05 vs. cells treated without domatinostat (i.e., at 0 nM). (**c**) Activation of the caspase pathway. Cells were treated with the indicated concentrations of domatinostat for 3 days and then subjected to Western blot analysis for the expression of the indicated proteins. Representative blots of two independent biological replicates are shown. The numbers below the Western blot images represent the means of the relative band intensities after each band was quantified by densitometry and normalized by the GAPDH value. * *p* < 0.05 vs. cells treated without domatinostat (i.e., at 0 nM).

Given the well-documented role of FOXM1 in cell cycle progression and cellular proliferation, in particular, of ovarian cancer cells [5,13], we next asked whether domatinostat, as an inhibitor of FOXM1 expression, exhibits growth inhibitory effects on ovarian cancer cells. To this end, we first determined the viability of cells after domatinostat treatment metabolically using the WST-8 assay. Whereas domatinostat up to 500 nM did not affect the viability of normal human fibroblasts (IMR90), it inhibited the viability of ovarian cancer cell lines in a concentration-dependent manner within the concentration range (Figure 2a). Subsequently, the results of the propidium iodide incorporation assay indicated that induction of cell death was involved in the growth inhibitory effects of domatinostat on ovarian cancer cells (Figure 2b). Since domatinostat-induced ovarian cancer cell death was accompanied by caspase activation (Figure 2c), the results suggested that domatinostat inhibited the growth and survival of ovarian cancer cells at least in part by induction of apoptosis.

### 2.3. Domatinostat Reduces the Expression of Survivin Essential for the Survival of Ovarian Cancer Cells

**Figure 3:**
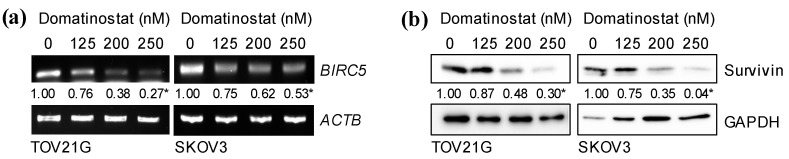
Domatinostat reduces the mRNA and protein expression of survivin in ovarian cancer cells. Ovarian cancer cells treated with the indicated concentrations of domatinostat were subjected to RT-PCR (**a**) or Western blot (**b**) analysis for *BIRC5*/survivin expression. Representative images of two independent biological replicates are shown. The numbers below the RT-PCR and Western blot images represent the means of the relative band intensities after each band was quantified by densitometry and normalized by the *ACTB* (RT-PCR) or the GAPDH (Western blot) value. * *p* < 0.05 vs. cells treated without domatinostat (i.e., at 0 nM).

**Figure 4:**
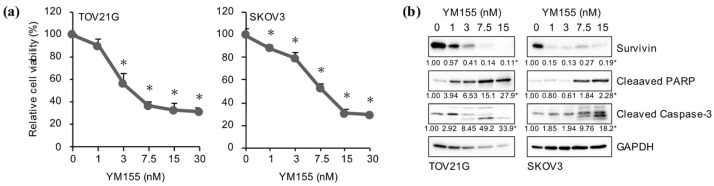
Reduction of survivin expression is sufficient to activate the caspase pathway and reduce the viability of ovarian cancer cells. (**a**) Ovarian cancer cells treated with the indicated concentrations of YM155 for 3 days were subjected to the WST-8 assay. Values are presented as the means + SD of triplicate samples. * *p* < 0.05 vs. cells treated without YM155 (i.e., at 0 nM). (**b**) Ovarian cancer cells were treated with the indicated concentrations of YM155 for 3 days and then subjected to Western blot analysis for the expression of the indicated proteins. Shown are representative images of two independent biological replicates. The numbers below the Western blot images represent the means of the relative band intensities after each band was quantified by densitometry and normalized by the GAPDH value. * *p* < 0.05 vs. cells treated without YM155 (i.e., at 0 nM).

FOXM1 is known to have a variety of transcriptional targets that mediate its pleiotropic cellular functions, among which is *BIRC5*, the gene encoding the anti-apoptotic protein survivin [14,15,16]. Since domatinostat reduced the expression of FOXM1 in ovarian cancer cells, we surmised that survivin may be involved in domatinostat-induced apoptosis of ovarian cancer cells. To test this idea, we examined the mRNA and protein levels of survivin in ovarian cancer cells after domatinostat treatment. Domatinostat inhibited not only protein but also mRNA expression of survivin (Figure 3a,b), in support of the idea that domatinostat inhibits FOXM1-mediated transcription of the *BIRC5* gene. To determine whether the transcription of the *BIRC5* gene was actually required for the survival of the ovarian cancer cells, we treated them with YM155, a pharmacological inhibitor of survivin expression that blocks the promoter activity of the *BIRC5* gene [17,18]. YM155 inhibited survivin expression, activated the caspase pathway and reduced the viability of ovarian cancer cells (Figure 4a,b), suggesting that the endogenous expression of survivin in ovarian cancer cells was required to prevent them from undergoing apoptosis and that domatinostat induced apoptosis in ovarian cancer cells by targeting survivin expression.

### 2.4. FOXM1 Is Required to Maintain Survivin Expression in Ovarian Cancer Cells

**Figure 5:**
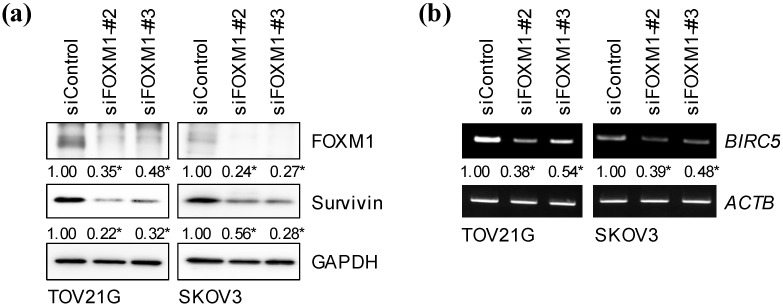
Survivin expression is dependent on FOXM1 in ovarian cancer cells. Ovarian cancer cells were transiently transfected with either an siRNA against FOXM1 (siFOXM1 #2, #3) or control RNA (siControl). After 3 days, the transfected cells were subjected to Western blot (**a**) and RT-PCR (**b**) analyses for the indicated proteins and mRNA, respectively. Representative images of at least two independent biological replicates are shown. The numbers below the Western blot and the RT-PCR images represent the means of the relative band intensities after each band was quantified by densitometry and normalized by the GAPDH (Western blot) or the *ACTB* (RT-PCR) value. * *p* < 0.05 vs. cells transfected with siControl.

We next confirmed whether the reduced survivin expression in ovarian cancer cells after domatinostat treatment was attributable to its inhibition of FOXM1 expression. To this end, we knocked down FOXM1 in ovarian cancer cells and examined its impact on survivin expression. Two different siRNAs targeting different regions of FOXM1 mRNA, both of which reduced the expression of FOXM1 albeit with different efficiencies, were used to knockdown FOXM1 (Figure 5a). Importantly, we were able to confirm that FOXM1 knockdown led to reduced viability of ovarian cancer cells, just as reported previously [13]. We then examined the expression levels of survivin after FOXM1 knockdown and observed reduced expression of survivin upon silencing of FOXM1 (Figure 5a), which demonstrated that survivin expression was dependent on FOXM1 in ovarian cancer cells. FOXM1 knockdown also reduced the mRNA levels of survivin/*BIRC5* (Figure 5b), suggesting that the *BIRC5* gene was a transcriptional target of FOXM1 in ovarian cancer cells just as in other cell types reported previously [14,15,16]. Collectively, the results presented so far in this study suggested that domatinostat reduced the viability of ovarian cancer cells by targeting the FOXM1-survivin axis.

### 2.5. Domatinostat Combined with Cisplatin and Paclitaxel Effectively Inhibits the Viability of Ovarian Cancer Cells

**Figure 6:**
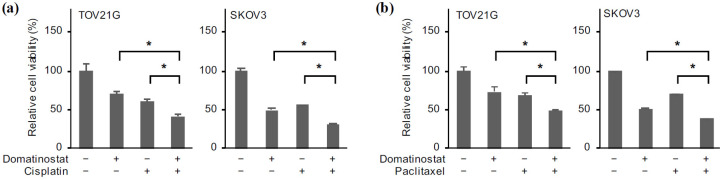
Domatinostat reduces the viability of ovarian cancer cells in combination with cisplatin and paclitaxel. Ovarian cancer cells treated without or with domatinostat (200 nM) in the absence or presence of cisplatin (1 μM for TOV21G, 2 μM for SKOV3) (**a**) and paclitaxel (2 nM) (**b**) for 3 days were subjected to the WST-8 assay. The values represent the means + SD obtained from at least triplicate samples. * *p* < 0.05. Similar results were obtained from three independent biological replicates.

Chemoresistance is a key characteristic of ovarian cancer that adversely affects its prognosis [19,20,21]. Mounting evidence suggests that FOXM1 overexpression contributes to chemoresistance in ovarian cancer [5], while it has been well established that survivin confers chemoresistance on cancer cells in general by increasing the threshold for chemotherapy-induced apoptosis [22,23,24,25]. We therefore examined next whether domatinostat, as an inhibitor of both FOXM1 and survivin expression, could be effectively combined with platinum- and taxane-based chemotherapy. The results indicated that treatment of ovarian cancer cells with either domatinostat or cisplatin alone, each at its clinically relevant concentration, reduced the viability of ovarian cancer cells and that the combination of both reduced the viability significantly more effectively than either alone (Figure 6a). Essentially similar results were obtained when paclitaxel was used instead of cisplatin (Figure 6b). Together, the results indicated that cisplatin and paclitaxel inhibited the viability of ovarian cancer cells more effectively in combination with domatinostat.

### 2.6. Reduced Survivin Expression Mimics the Effects of Domatinostat

**Figure 7:**
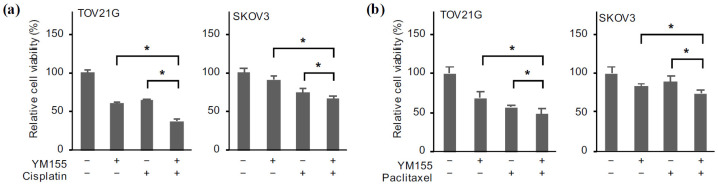
Pharmacological inhibition of survivin expression is sufficient to reduce the viability of ovarian cancer cells in combination with cisplatin and paclitaxel. Ovarian cancer cells treated without or with YM155 (3 nM) in the absence or presence of cisplatin (1 μM for TOV21G, 2 µM for SKOV3) (**a**) and paclitaxel (2 nM) (**b**) for 3 days were subjected to the WST-8 assay. The values are shown as the means + SD obtained from at least triplicate samples. * *p* < 0.05. Similar results were obtained from three independent biological replicates.

**Figure 8:**
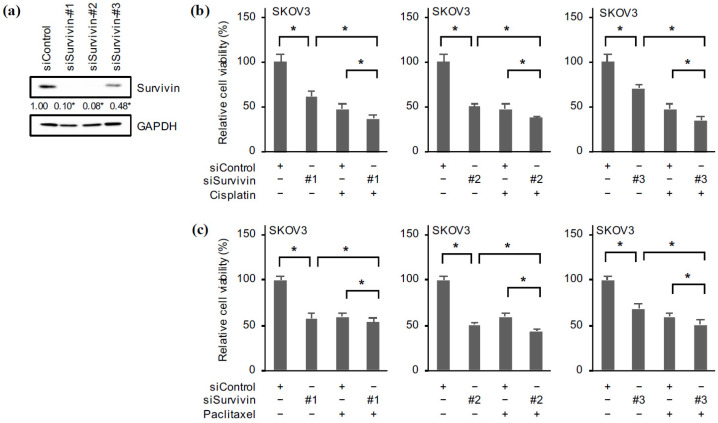
Genetic silencing of survivin reduces the viability of ovarian cancer cells alone and in combination with cisplatin and paclitaxel. (**a**) SKOV3 cells transiently transfected with an siRNA against survivin (siSurvivin #1–#3) or control siRNA (siControl) for 4 days were subjected to Western blot analysis for survivin expression. Representative blots of two independent biological replicates are shown. The numbers below the Western blot images represent the means of the relative band intensities after each band was quantified by densitometry and normalized by the GAPDH value. * *p* < 0.05 vs. cells transfected with siControl. Alternatively, 24 h after transfection, cells were further treated with 2.5 µM cisplatin (**b**) or 4 nM paclitaxel (**c**) for 3 days and then subjected to the WST-8 assay. The values are shown as the means + SD obtained from at least triplicate samples. * *p* < 0.05. Similar results were obtained from three independent biological replicates.

While emerging lines of evidence from recent studies using genetic knockdown and pharmacological inhibitors have clearly delineated the crucial role of FOXM1 in the chemoresistance of ovarian cancer [5], the role of survivin in the chemoresistance of ovarian cancer in particular remains less well characterized. We therefore wished to determine whether the reduced survivin expression after domatinostat treatment contributed to the enhanced effects of domatinostat in combination with cisplatin and paclitaxel. When ovarian cancer cells were treated with these chemotherapeutic agents (cisplatin and paclitaxel) in the absence and presence of YM155, their viability was more effectively reduced in the presence of YM155, suggesting that pharmacological inhibition of survivin expression enhanced the growth inhibitory effects of cisplatin and paclitaxel (Figure 7a,b). The results were confirmed by genetic knockdown of survivin in SKOV3 cells using three different siRNAs against survivin (Figure 8a). Not only did survivin knockdown by itself reduce the viability of ovarian cancer cells in support of the earlier results obtained by using YM155 (Figure 4a), the combination of survivin knockdown and cisplatin treatment also reduced cell viability more effectively than either alone (Figure 8b). The effect of paclitaxel on cell viability was also enhanced when paclitaxel treatment was combined with survivin knockdown (Figure 8c). These results suggested that survivin expression was required for the survival and chemoresistance of ovarian cancer cells and, in turn, that domatinostat reduced the viability and increased the chemosensitivity of ovarian cancer cells through downregulation of survivin expression.

## 3. Discussion

Since the seminal identification of the aberrant overexpression and activation of the transcription factor FOXM1 as a key molecular alteration in ovarian cancer, a large body of evidence has documented the pivotal role played by FOXM1 in the malignant phenotypes of ovarian cancer, such as enhanced cell proliferation, survival, invasion, and chemoresistance [4,5]. As such, FOXM1 is attracting ever-increasing attention as a viable therapeutic target to overcome the aggressive nature of ovarian cancer. Here in this study, we tested the possibility of domatinostat, a class I-selective HDAC inhibitor in clinical and preclinical stages of development for the treatment of cancer, as an inhibitor of FOXM1 expression in ovarian cancer cells. Our results clearly showed that domatinostat inhibited the expression of FOXM1 in ovarian cancer cells at clinically relevant concentrations and accordingly reduced their viability and enhanced the growth inhibitory effects of standard ovarian cancer chemotherapeutic agents cisplatin and paclitaxel. Furthermore, we showed that domatinostat inhibits the expression of *BIRC5*/survivin, a transcriptional target of FOXM1, along with FOXM1, and that pharmacological or genetic inhibition of survivin was sufficient to mimic the growth inhibitory and chemosensitizing effects of domatinostat. Although the data alone may not necessarily eliminate the possibility that domatinostat exerted its growth-inhibitory and chemosensitizing effects also through other mechanisms independent of survivin, our results suggest that the ability of domatinostat to target the FOXM1–survivin axis underlies its anti-cancer activities in ovarian cancer cells.

Domatinostat is a small molecule identified and characterized as a selective inhibitor of class I HDACs and has shown anti-cancer activities in vitro and in vivo against a variety of malignancies, both hematological and non-hematological, in preclinical studies [12,26,27]. Importantly, a phase 1 study conducted in 24 patients with treatment-refractory, advanced hematological malignancies demonstrated not only acceptable tolerability and safety of domatinostat but also promising signs of anti-tumor activity, with one patient achieving a complete response, another achieving a partial response, and 18 having stable disease [9]. Given that the IC_50_ values for ovarian cancer cell lines determined in the present study were quite comparable to those for a panel of cell lines from hematological malignancies [9], domatinostat may be an excellent candidate as a therapeutic for ovarian cancer patients and thus warrants preclinical studies to test its therapeutic potential in animal models of ovarian cancer.

Currently, it remains unknown how domatinostat inhibits the expression of FOXM1. Since HDACs are known to target not only histones but also non-histone substrates [28,29], domatinostat, as an inhibitor of class I HDACs, may interact with the regulatory mechanism of FOXM1 expression either epigenetically or non-epigenetically. An alternative, intriguing possibility behind domatinostat inhibition of FOXM1 is that lysine-specific demethylase 1 (LSD1) is involved in the inhibition of FOXM1 expression as well as in the anti-cancer activities of domatinostat. Besides class I HDACs, domatinostat has been shown to inhibit the activity of LSD1 [9,26,30]. Interestingly, previous studies investigating the role of LSD1 in ovarian cancer consistently showed that LSD1 was overexpressed in ovarian cancer and that LSD1 overexpression was associated with poor survival of ovarian cancer patients [31,32]. Furthermore, not only did inhibition of LSD1 in ovarian cancer cells, either pharmacologically or genetically, reduce the viability of ovarian cancer cells [31,33], LSD1 knockdown resulted in reduced expression of survivin [33]. Although the effect of LSD1 inhibition on FOXM1 expression was not investigated in the study, these findings together suggest the possibility that LSD1 plays an essential role in the growth and survival of ovarian cancer cells through the activation of the FOXM1–survivin axis. Recently, with the increased awareness of the role played by LSD1 in various pathological conditions including cancer, an increasing number of LSD1 inhibitors have been developed and are entering the clinical trial stage [34]. It would therefore be of interest to examine such newly developed LSD1 inhibitors for their ability to inhibit FOXM1 expression as well as for their anti-cancer activity against ovarian cancer cells in future studies.

Reflecting its well-established role in the chemoresistance of various human cancers including ovarian cancer, FOXM1 has a variety of transcriptional targets in addition to *BIRC5*/survivin that can contribute to the chemoresistance phenotype at multiple levels such as DNA repair, drug efflux, and microtubule dynamics [8]. It is therefore quite natural to assume that these transcriptional targets of FOXM1 other than *BIRC5*/survivin also played some roles in the combinational effects of domatinostat with cisplatin and paclitaxel. Since survivin is supposed to directly bind and inhibit initiator and effector caspases [22,25], the reduced expression of survivin in ovarian cancer cells after domatinostat treatment is considered to have contributed to the anti-cancer activities of domatinostat by lowering the threshold for apoptosis. Notably, our results indicated that the pharmacological inhibitor of survivin, YM155, effectively reduced survivin expression and the viability of ovarian cancer cells alone or in combination with cisplatin and paclitaxel, suggesting that YM155 itself may be promising as a therapeutic for ovarian cancer. However, while YM155 is recognized as a highly potent inhibitor of survivin expression in vitro, it exhibited very limited anti-tumor efficacy when used alone or in combination with chemotherapeutic agents in multiple phase 1 and phase 2 clinical trials, which can most likely be ascribed to the chemical instability of YM155 [35]. In this regard, the pharmacokinetic analysis of domatinostat in the phase 1 study showed that oral administration of domatinostat resulted in measurable plasma concentrations of domatinostat, which were sufficient to show signs of anti-tumor activity [9].

In summary, we have shown in this study that domatinostat, at clinically relevant concentrations, reduces the expression of FOXM1 and its transcriptional target survivin, and accordingly the viability of ovarian cancer cells alone and in combination with chemotherapeutic agents. Our results thus suggest that domatinostat, as an inhibitor of FOXM1 expression, may become an attractive and viable treatment option for ovarian cancer.

## 4. Materials and Methods

### 4.1. Reagents and Antibodies

Domatinostat (4SC-202; S7555) was purchased from Selleck Chemicals (Houston, TX, USA). Domatinostat was dissolved in DMSO to prepare a 10 mM stock solution. Propidium iodide (P3566) and Hoechst33342 (H3570) solutions were purchased from Thermo Fisher Scientific Inc. (Waltham, MA, USA). An antibody against FOXM1 (ab207298) was purchased from Abcam plc (Cambridge, UK). Antibodies against Survivin (#2808), GAPDH (#5174), cleaved PARP (#9541), and cleaved caspase 3 (#9661) were purchased from Cell Signaling Technology Inc. (Beverly, MA, USA). Horseradish peroxidase (HRP)-conjugated anti-rabbit IgG and anti-mouse IgG secondary antibodies were purchased from Jackson ImmunoResearch (West Grove, PA, USA).

### 4.2. Cell Culture

TOV21G, an ovarian clear cell cancer cell line [36,37], and SKOV3, an ovarian serous cancer cell line [38,39], were purchased from the American Type Culture Collection (ATCC, Manassas, VA, USA) and cultured in DMEM/F-12 medium supplemented with 10% fetal bovine serum (FBS), 100 units/mL of penicillin, and 100 μg/mL of streptomycin. IMR90, a human normal fetal lung fibroblast cell line, was purchased from ATCC and maintained in DMEM supplemented with 10% FBS, 100 units/mL of penicillin, and 100 μg/mL of streptomycin. All IMR90 experiments were performed using cells with a low passage number (<8).

### 4.3. Cell Viability Assay

Cell viability was evaluated using the WST-8 assay [40,41]. Cells plated in a 96-well plate (0.5–1 × 10^4^/well) were treated as described in the figure legends. WST-8 reagent (Cell Counting Kit-8, DOJINDO Laboratories, Kumamoto, Japan) was then added and cells were incubated at 37 °C for 1–3 h. Absorbance at 450 nm was measured using a microplate reader (iMark; Bio-Rad Laboratories, Inc., Hercules, CA, USA). Relative cell viability was calculated as a percentage of the absorbance of treated samples relative to that of controls.

### 4.4. Propidium Iodide (PI) Incorporation Assay

To assess cell death, the PI incorporation assay was used [42,43]. Cells treated as indicated in the figure legend were incubated with PI (1 μg/mL) and Hoechst33342 (10 μg/mL) for 5 min at 37 °C. To calculate the ratio of PI-positive cells (dead cells) to Hoechst-positive cells (total cells), fluorescent images were obtained using a fluorescence microscope equipped with iPhone (CKX41; Olympus, Tokyo, Japan) and scored. More than 130 cells were counted to calculate the percentage of PI-positive cells.

### 4.5. Western Blot Analysis

Western blot analysis was conducted as previously described [41,44]. Cells were harvested and washed with ice-cold PBS and lysed in RIPA buffer (10 mM Tris/HCl (pH 7.4), 1% Nonidet P-40, 0.1% sodium dodecyl sulfate (SDS), 0.1% sodium deoxycholate, 150 mM NaCl, 1 mM EDTA, 10 mM sodium pyrophosphate, 1.5 mM sodium orthovanadate, 10 mM sodium fluoride, and protease inhibitor cocktail set III (FUJIFILM Wako Chemicals, Osaka, Japan). The lysates were immediately mixed with the same volume of 2× Laemmli buffer (125 mM Tris/HCl (pH 6.8), 4% SDS, and 10% glycerol) and boiled at 95 °C for 10 min. After the protein concentrations of cell lysates were measured using a BCA protein assay kit (Thermo Fisher Scientific Inc., Waltham, MA, USA), samples containing equal amounts of protein were separated by SDS/polyacrylamide gel electrophoresis and transferred to polyvinylidene difluoride membranes. Membranes were probed with the indicated primary antibodies followed by appropriate HRP-conjugated secondary antibodies as recommended by the manufacturer of each antibody. Immunoreactive bands were visualized using Immobilon Western Chemiluminescent HRP Substrate (Merck KGaA, Darmstadt, Germany) and detected by a ChemiDoc Touch device (Bio-Rad Laboratories, Inc., Hercules, CA, USA). Quantification of the bands on the membranes was performed by densitometry using ImageJ software (version 1.53k) (http://imagej.nih.gov/ij/, accessed on 4 April 2020).

### 4.6. Reverse Transcription (RT)-PCR Analysis

RT-PCR analysis was conducted as previously described [41]. Total RNA was extracted from cells using Trizol (Thermo Fisher Scientific Inc.), and 500 ng of total RNA was reverse transcribed using the PrimeScript RT reagent kit (Takara Bio Inc., Shiga, Japan) according to the manufacturer’s protocol. RT-PCR analysis was performed with the following primers: *FOXM1*, 5′-CCAGGGTCTCCACAATTGCC-3′ (forward) and 5′-CTCAGCTAGCAGCACTGATAAAC-3′ (reverse); *BIRC5*, 5′-CCTTTCTCAAGGACCACCGCATC-3′ (forward) and 5′-CGTCATCTGGCTCCCAGCCTT-3′ (reverse); *ACTB*, 5′-CCCATGCCATCCTGCGTCTG-3′ (forward) and 5′-CGTCATACTCCTGCTTGCTG-3′ (reverse). Target genes were amplified with Quick Taq HS DyeMix (Toyobo Co., Ltd., Osaka, Japan). Quantification of the bands in the gels was performed by densitometry using ImageJ software (version 1.53k) (http://imagej.nih.gov/ij/, accessed on 4 April 2020).

### 4.7. Gene Silencing by siRNA

siRNAs against human *FOXM1* (#2: HSS103713 and #3: HSS177135), *survivin*/*BIRC5* (#1: HSS179403, #2: HSS179404 and #3:HSS179405), and control RNA (Stealth RNAi siRNA negative control medium GC duplex #2) were purchased from Thermo Fisher Scientific Inc. Cells were transiently transfected with one of the siRNAs against *FOXM1* (siFOXM1; 120–200 pmol per 6 cm dish) or *survivin*/*BIRC5* (siSurvivin; 120–200 pmol per 6 cm dish) or with the control RNA (siControl; 120–200 pmol per 6 cm dish) using Lipofectamine RNAiMAX (Thermo Fisher Scientific Inc.) according to the manufacturer’s instructions.

### 4.8. Statistical Analysis

Results are shown as means + standard deviation (SD). Data were analyzed using the Student’s *t*-test for comparisons between two groups. Differences with a *p*-value < 0.05 were considered to be significant and are indicated with asterisks in the figures.

## Data Availability

All data are contained in this article and there are no repository data.
